# Requirement of Gαi1 and Gαi3 in interleukin-4-induced signaling, macrophage M2 polarization and allergic asthma response

**DOI:** 10.7150/thno.56383

**Published:** 2021-03-04

**Authors:** Jin-yu Bai, Ya Li, Guan-hua Xue, Ke-ran Li, Yu-Fan Zheng, Zhi-qing Zhang, Qin Jiang, Yuan-yuan Liu, Xiao-zhong Zhou, Cong Cao

**Affiliations:** 1 Department of Orthopedics, the Second Affiliated Hospital of Soochow University, Suzhou, China.; 2 Jiangsu Key Laboratory of Neuropsychiatric Diseases and Institute of Neuroscience, Soochow University, Suzhou, China.; 3 The Central Lab, North District, The Affiliated Suzhou Hospital of Nanjing Medical University, Suzhou Municipal Hospital, Suzhou, China.; 4 Department of Vascular Surgery, Renji Hospital, School of Medicine, Shanghai Jiaotong University, Shanghai, China.; 5 The Fourth School of Clinical Medicine, The Affiliated Eye Hospital, Nanjing Medical University, Nanjing, China.; 6 Clinical research & lab center, Affiliated Kunshan Hospital of Jiangsu University, Kunshan, China.

**Keywords:** IL-4, Gαi1/3, M2 polarization, allergic asthma response, signaling

## Abstract

IL-4 induces Akt activation in macrophages, required for full M2 (alternative) polarization. We examined the roles of Gαi1 and Gαi3 in M2 polarization using multiple genetic methods.

**Methods and Results:** In MEFs and primary murine BMDMs, Gαi1/3 shRNA, knockout or dominant negative mutations attenuated IL-4-induced IL4Rα endocytosis, Gab1 recruitment as well as Akt activation, leaving STAT6 signaling unaffected. Following IL-4 stimulation, Gαi1/3 proteins associated with the intracellular domain of IL-4Rα and the APPL1 adaptor, to mediate IL-4Rα endosomal traffic and Gab1-Akt activation in BMDMs. In contrast, gene silencing of Gαi1/3 with shRNA or knockout resulted in BMDMs that were refractory to IL-4-induced M2 polarization. Conversely, Gαi1/3-overexpressed BMDMs displayed preferred M2 response with IL-4 stimulation. In primary human macrophages IL-4-induced Akt activation and Th2 genes expression were inhibited with Gαi1/3 silencing, but augmented with Gαi1/3 overexpression. In Gαi1/3 double knockout (DKO) mice, M2 polarization, by injection of IL-4 complex or chitin, was potently inhibited. Moreover, in a murine model of asthma, ovalbumin-induced airway inflammation and hyperresponsiveness were largely impaired in Gαi1/3 DKO mice.

**Conclusion:** These findings highlight novel and essential roles for Gαi1/3 in regulating IL-4-induced signaling, macrophage M2 polarization and allergic asthma response.

## Introduction

In response to environmental stimuli, macrophages are polarized into pro-inflammatory M1 (classical) and immunomodulatory M2 (alternative) subtypes 1, 2. Lipopolysaccharide (LPS), interferon-γ (IFN-γ) and other stimuli lead to M1 activation, inducing pro-inflammatory cytokine production and inflammatory responses 1, 2. Alternatively, the Th2 cytokine IL-4 elicits M2 macrophage production, inducing helminthic immunity, allergy, and other immunomodulatory activities 3-7.

M2 polarization is driven by two major signaling pathways, JAK1/STAT6 and PI3K/AKT, following IL-4 receptor (IL-4R) stimulation of macrophages. Activation of IL-4 receptor (IL-4R) on the surface of macrophages is required for the activation of STAT6 (signal transducer and activator of transcription 6), a key transcription factor for M2 polarization 8, 9. In response to IL-4, STAT6 and nuclear receptors, including peroxisome proliferator-activated receptorγ (PPARγ) and PPARδ, are activated in M2 macrophages 10, 11. This increases expression of the prototypic M2 marker *Arginase-1*, promoting L-arginine-L-ornithine exchange, polyamine synthesis and tissue repair 12. M2 macrophages also show significantly upregulated C-type lectins, mannose receptor, chitinase family proteins, resistin-like molecules, and interleukin-10 (IL-10), to exert immunomodulatory functions 1, 2. However, the underlying molecular mechanisms of M2 polarization remain unclear.

Recent studies have highlighted a pivotal role for Akt-mTOR signaling in M2 polarization 13, 14. IL-4-induced Akt activation, independent of STAT6 signaling, is required for full M2 activation 14. Akt inhibition significantly inhibited IL-4-induced M2 polarization in macrophages 14. Conversely, Akt activation following mTOR inhibition rescues defective M2 polarization 13.

The present study examined the roles of Gαi1 and Gαi3 (Gαi1/3) in mediating IL-4-induced Akt-mTOR signaling. Gαi1/Gαi3 are heterotrimeric G proteins that play critical roles in mediating the PI3K-Akt-mTOR and Erk signaling pathways to serve non-canonical functions for signal transduction of multiple receptor tyrosine kinases (RTKs) 15-19. The inhibitory subunit of the heterotrimeric guanine nucleotide-binding proteins, Gαi proteins, have three primary members, Gαi1, Gαi2, and Gαi3 20. The coupling of Gαi proteins with G protein coupled receptors (GPCRs) can repress adenylate cyclase (AC) activity to suppress cyclic AMP (cAMP) production 20. Following stimulation of RTKs, Gαi1/3 are recruited to mediate PI3K-Akt-mTOR and Erk signal transduction 15-19. Our results here indicate that Gαi1/3 are required for IL-4-induced Akt signaling activation and M2 polarization in macrophages.

## Methods

### Ethics

Protocols of this study were approved by the Ethics Committee of Soochow University.

### Materials and reagents

IL-4, puromycin, pertussis toxin (PTX) and polybrene were purchased from Sigma-Aldrich (St. Louis, Mo). Fetal bovine serum (FBS) and other reagents for cell culture were purchased from Gibco BRL (Grand Island, NY). From Cell Signaling Tech (Shanghai, China) and Santa Cruz Biotech (Santa Cruz, CA) the antibodies were purchased. The constitutively active Akt1 (caAkt1) adenovirus (“Ad-caAkt1”) was reported early 21.

### Mouse embryonic fibroblasts (MEFs)

As reported early 15, 16, 18, 19, wild-type (WT), Gαi1 and Gαi3 doubly knockout (DKO), Gαi1, Gαi2 or Gαi3 single knockout (SKO) MEFs, as well as WT and Grb2-associated binder-1 (Gab1) KO MEFs were cultured in FBS-containing DMEM medium. MEFs were starved in 0.5% FBS medium overnight and 30 min in warm PBS before any treatment.

### Murine BMDMs

The bone marrow of WT and Gαi1/3 DKO mice 15, 16 were flushed by complete RPMI medium (with FBS), with the resulting cell pellets resuspended in ACK hypotonic buffer. The remaining bone marrow cells were washed with complete RPMI medium, and cultured in RPMI medium with 30% L-929 cell medium 22. Within 8-10 days the adherent primary bone marrow-derived macrophages (BMDMs) were trypsinized, washed and re-plated for the further experimental usage.

### Gαi1/3 shRNA

At 100, 000 cells per well, MEFs or BMDMs were seeded into six-well tissue culture plates, and Gαi1 shRNA lentivirus and/or the Gαi3 shRNA lentivirus 15, 16 were added. The culture medium was replaced with fresh puromycin-containing culture medium every two days, until resistant colonies were formed (10-12 days). In stable cells Gαi1/3 knockdown (over 90% knockdown efficiency) was verified by Western blotting and quantitative real-time PCR (qPCR).

### CRISPR/Cas9 knockout of Gαi1 and Gαi3

The lentiviral CRISPR/Cas-9 Gαi1 KO construct and lentiviral CRISPR/Cas-9 Gαi3 KO construct were designed and purchased from Shanghai Genechem (Shanghai, China) 15, transfected into MEFs/BMDMs, and selected with puromycin. Control cells were treated with the empty vector with control sgRNA (Santa Cruz Biotech). In stable cells Gαi1/3 knockout was confirmed by Western blotting and qPCR.

### Gαi1/3 overexpression or mutation

At 100, 000 cells per well, BMDMs or human macrophages were seeded into six-well tissue culture plates, murine/human Gαi1-expressing adenovirus (Ad-Gαi1) and murine Gαi3-expressing adenovirus (Ad-Gαi3) 15, 16 were added. Cells were selected by puromycin. Control cells were treated with the empty vector-expressing adenovirus. In stable cells Gαi1/3 overexpression was confirmed by Western blotting and qPCR. The dominate negative Gαi1 construct and the dominate negative Gαi3 construct were described in our previous study, co-transfected to cultured BMDMs 19.

### Generation of the Gαi1/3 double knock (DKO) mice

The generation of Gαi1/3 DKO mice by the CRISPR-Cas9 method was described previously 15, 16. This study was performed in strict accordance with the recommendations in the Guide for the Care and Use of Laboratory Animals of the National Institutes of Health. All of the animals were handled according to approved institutional animal care and use committee (IACUC) protocols of Soochow University. The protocols was approved by the Committee on the Ethics of Animal Experiments of Soochow University.

### qPCR

The detailed protocols for qPCR by the ABI-7600 Prism equipment and the SYBR Green PCR kit were previously described 15, 23. mRNA expression of targeted genes was quantified via the the ^ΔΔ^Ct protocol. Samples from BMDMs were normalized to hypoxanthine phosphoribosyltransferase (HPRT) and samples from PECs were normalized to the macrophage marker CD68. The primers were reported early 14. Other primers were synthesized and verified by Genechem Co. (Shanghai, China).

Western blotting and data quantification, co-immunoprecipitation (IP) assay, CCK-8 viability assay, and confocal microscopy were described in detail in our previous studies 16, 18, 19, 24. For all the Western blotting assay, each lane of a SDS-PAGE Gel was loaded with exact same amount of quantified protein lysates (40 μg in each treatment), the same set of lysate samples were run in parallel (“sister”) gels to test different proteins (same for all Figures).

### Plasma membrane fractionation

The detailed protocols for plasma membrane isolation was described previously 15, 16.

### Endosome fractions

BMDMs with the applied treatments were harvested and re-suspended in the hypotonic swelling buffer 25, and lysed with 30 strokes in a Dounce homogenizer using a tight pestle, and swelling was stopped by the addition of two fold homogenization buffer 25. Lysates were centrifuged to obtain the post-nuclear supernatants, which was further centrifuged 25. The resulting supernatants were centrifuged, and the pellet solubilized in the homogenization buffer 25. Insoluble particles were removed by short centrifugation and the supernatant loaded onto a 5-20% continuous OptiprepTM (Sigma-Aldrich), poured using homogenization buffer. The gradient was further centrifuged at 60,000 g for 24h, with total 10 endosomal fractions collected, and proteins precipitated with 12% TCA for 1h. Fractions were centrifuged at 12,000 g for 1h. The protein pellets, combining all ten endosomal fractions, were dissolved in SDS-sample buffer for analysis by Western blotting.

### APPL1 overexpression or silencing

Briefly, the full-length mouse APPL1 (provided by Genechem, Shanghai, China) was sub-cloned into the GV248 construct, and transfected into human embryonic kidney 293 (HEK-293) cells, together with the lentivirus packaging plasmids (pCMV-VSVG and pCMV-ΔA.9, Genechem). The generated lentivirus was filtered, enriched and added to cultured BMDMs. Stable BMDMs were then established by culturing in the puromycin-containing medium. The murine APPL1 shRNA lentiviral particles (sc-61981-V) were provided by Santa Cruz Biotech (Beijing, China), added to cultured BMDMs (cultured in polybrene medium). Stable BMDMs were again established by puromycin selection. APPL1 expression was always verified by Western blotting assays in the stable BMDMs.

### CRISPR/Cas9 knockout of IL-4Rα

The murine IL-4Rα CRISPR/Cas9 KO construct (sc-421111) was purchased from Santa Cruz Biotech (Beijing, China). The plasmid was transfected to BMDMs via Lipofectamine 2000 (Thermo-Fisher, Invitrogen, Shanghai, China). Single BMDMs were cultured for two weeks, subjected to screen of IL-4Rα KO by qPCR and Western blotting assays. Stable BMDMs with complete depleted IL-4Rα were utilized for further experiments.

### Expression of lL-4Rα

The DNA fragments encoding the full-length mlL-4Rα or the intracellular domain-depleted mlL-4Rα (using the described primers 26, 27) were individually sub-cloned into the Xhol/HiridlII site of the pDC3.1-Flag plasmid (Genechem) to produce pDC3.1-mlL-4Rα-fused with with Flag construct: IL-4Rα-WT-Flag/IL-4Rα-ΔIC-Flag. The construct was transfected intoHEK-293 cells by Lipofectamine 3000 (Thermo-Fisher, Shanghai, China), together with the lentivirus packaging plasmids (Genechem). The generated lentivirus was filtered, enriched and added to cultured BMDMs. Stable BMDMs were then established by culturing in the puromycin-containing medium.

### IL-4 complex administration

As described 14, 28 IL-4 (Peprotech, Rocky, NJ) was suspended at a concentration of 500 μg/mL and mixed with anti-mouse IL-4 (Abcam, Shanghai, China) at a molar ratio of 2:1 and incubated 2 min. IL-4 complex was then suspended in normal PBS (25μg/mL IL-4 plus 125 ug/mL anti-IL-4). For each mouse 200 μl of IL-4 complex (5 μg IL-4 and 25 μg anti-IL-4) was injected intraperitoneally on day-0 and day-2, and PECs were collected at day-4.

### Chitin administration

As described previously 29, chitin was washed and sonicated on ice. The dissolved chitin was filtered and diluted within PBS to a concentration of 4 μg/mL. For each mouse, 800 ng chitin (dissolved in 200 μL PBS) was injected intraperitoneally, after 48h PECs were collected.

### Primary human macrophages

Primary human monocyte-derived macrophages (MDM) were provided by Dr. Sun at Shanghai Pulmonary Hospital 30. Macrophages were obtained from CD14 magnetic bead-selected monocytes 31 from peripheral blood mononuclear cells (PBMCs) of written-informed consent healthy donors 31. The detailed protocols for primary human macrophage cultivation were previously described 31.

### Ovalbumin-induced mouse asthma model

WT or Gαi1/3 DKO mice were sensitized by intraperitoneal injection of ovalbumin (OVA, twice, one week apart, Sigma) 32. One week following the last sensitization, mice were anesthetized and challenged with OVA or PBS as described 32. Airway responsiveness, pulmonary inflammation and immunoglobulin synthesis were compared in wild-type and Gαi1/3 DKO mice sensitized and challenged with PBS or OVA. Three days after aspiration challenge, airway responsiveness to intravenous acetylcholine chloride (Ach) administration was determined using the described protocol 32. The number of inflammatory cells in bronchoalveolar Lavage (BAL) was determined. Lungs were also fixed and subjected to HE staining and Masson staining. Mouse lung tissues were digested and minced as reported 33. After lysis of red blood cells (RBCs), the dissociated cells were underlaid with 7.5 mL of lymphocyte separation medium (Sigma, Shanghai, China) and cells were centrifuged. From the middle layer the mononuclear cells were incubated in six-well plates for two hours 33. Thereafter, the adherent cells were alveolar macrophages.

### Statistical analysis

Numerical data and and histograms presented were expressed as means ± standard deviation (SD). Comparison between any two groups was by two-tailed unpaired Student t test. Multiple group comparison was performed by one-way analysis of variance (ANOVA) with post hoc Bonferroni test (data were all with normal distribution). Values of ***P*** less than 0.05 were considered statistically significant.

## Results

### Gαi1/3 are required for IL-4-induced Akt-mTOR activation in MEFs

**Figure 1:**
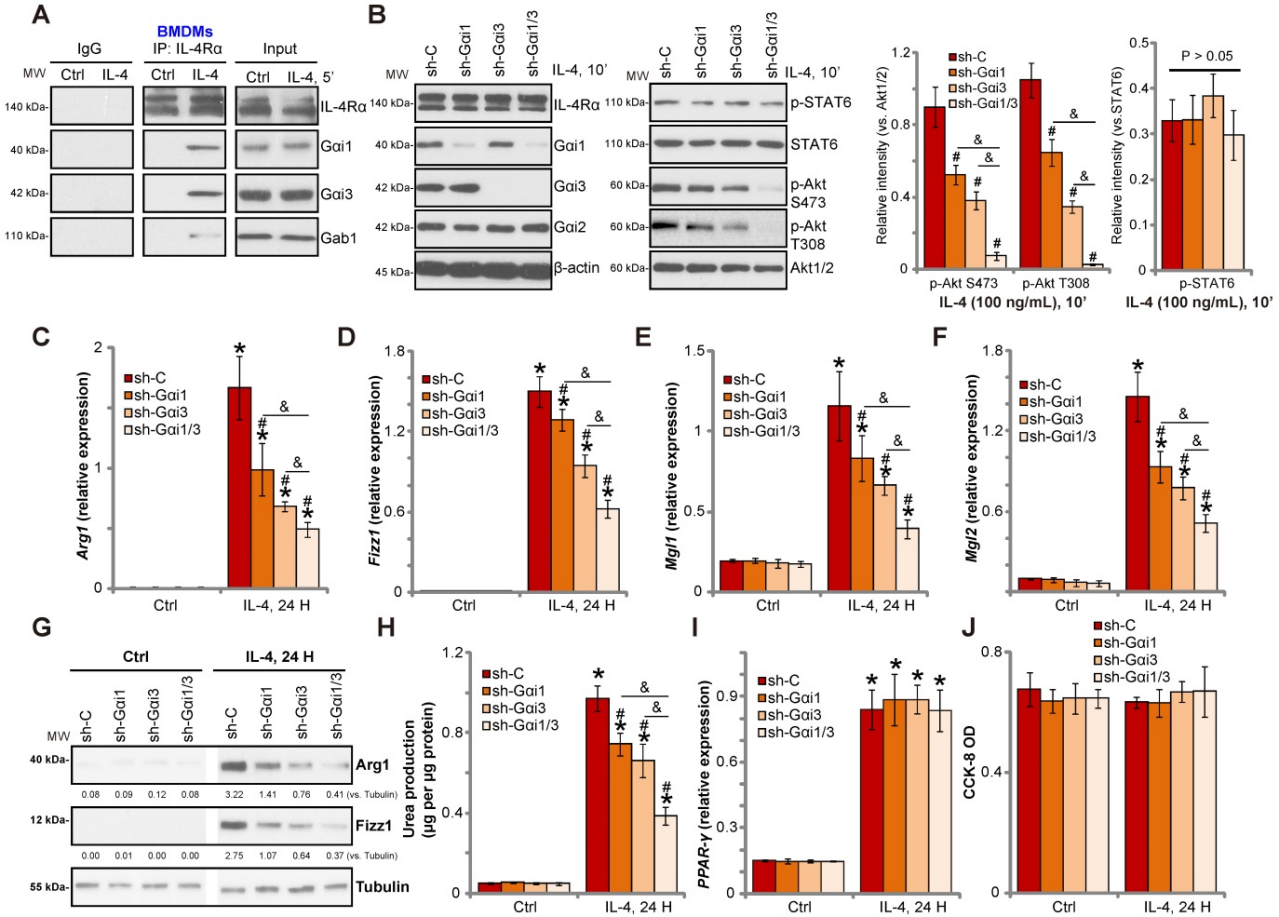
** Gαi1/3 knockdown inhibits IL-4-induced Akt activation and M2 polarization in BMDMs.** Primary cultured murine bone marrow-derived macrophages (BMDMs) were treated with IL-4 (100 ng/mL) for 5 min, IL-4Rα, Gαi1, Gαi3 and Gab1 association was tested by co-immunoprecipitation assay (**A**); Stable BMDMs, expressing the scramble control shRNA (“sh-C”), Gαi1 shRNA and/or Gαi3 shRNA, were treated with IL-4 (100 ng/mL) for applied time, and were tested by Western blotting of listed proteins (**B**); Relative expression of listed genes (24h after IL-4 treatment) was shown (**C**-**G**, and **I**); The urea production was also tested (**H**); Cell viability was tested by CCK-8 assay (**J**). For qPCR, Urea production and viability assays, in each experiment, n=5 (five replicated wells/dishes). Blotting quantification was performed from five replicate blot data (n=5, same for the blotting data in all Figures). Experiments were repeated three times (Same for all following Figures), data of all repeated experiments were pulled together to calculate mean ±SD (Same for all following Figures). “Ctrl” stands for untreated control. *P < 0.01 vs. “Ctrl” treatment in “sh-C” cells (**B**-**I**).**^ #^*P*** < 0.01 vs. IL-4 treatment in “sh-C” cells (**B**-**H**). ^&^***P*** < 0.01 (**B**-**G**).

As Gαi1/3 binds to RTKs to mediate downstream signal transduction 15-17, 19, we tested whether Gαi1/3 are important for IL-4-induced signaling. We first compared IL-4 signaling responses in wild-type (WT) and Gαi1 and Gαi3 double knockout (DKO) MEFs 15, 16, 18, 19. Total IL-4Rα, STAT6, Gαi2 expression, and IL-4-induced STAT6 phosphorylation were unchanged between WT and DKO MEFs (Figure **S1A**). In contrast, IL-4-induced phosphorylation of Akt (at both Ser-473 and Thr-308), p70S6K1 (“S6K1”, at Thr-389, the indicator of mTORC1 activation 34, 35) and Erk1/2 (Thr202/Tyr204) were significantly reduced in Gαi1/3 DKO MEFs (Figure **S1B**). Single knockout (SKO) of Gαi1 or Gαi3 resulted in partial inhibition of Akt, S6K and Erk1/2 phosphorylation in response to IL-4 (Figure **1C**), with Gαi3 SKO having a greater effect than Gαi1 SKO in suppressing Akt-mTOR and Erk activation (Figure **S1C**, quantification). Gαi2 SKO failed to significantly affect IL-4-induced Akt-S6K1 and Erk1/2 phosphorylation in MEFs (Figure **S1D**).

To confirm that loss of the Gαi-1/3 genes were responsible, rescue experiments were performed using an adenovirus Gαi1 construct (“Ad-Gαi1”, no Tag 15) or Gαi3 construct (“Ad-Gαi3”, no Tag 15) to exogenously express the proteins in the Gαi1/3 DKO MEFs. Following re-expression of Gαi1 or Gαi3, IL-4-induced phosphorylation of Akt-S6K and Erk1/2 were partially restored in DKO MEFs (Figure **S1E**). To further substantiate that Gαi1/3 are required for IL-4 signaling, we knocked down Gαi1 and Gαi3 using shRNA (see our previous studies 15, 16). Consistent with the above results, Gαi1/Gαi3 double knockdown blocked IL-4-induced phosphorylation of Akt, S6K and Erk1/2 (Figure **S1F**). Collectively, these results show that Gαi1/3 are required for IL-4-induced Akt-mTOR and Erk activation in MEFs.

### Gαi1 and Gαi3 are required for IL-4-induced IL-4Rα endocytosis and Gab1 recruitment/activation

It has been reported that IL-4 stimulation results in IL-4Rα endocytosis 26, 27, 36. Our previous studies have found that Gαi1 and Gαi3 are required for the endocytosis of VEGFR2 (the VEGF receptor) 15 and TrkB (the BDNF receptor) 16. Here, we showed that IL-4-induced IL-4Rα endocytosis was blocked in Gαi1/3 DKO MEFs, indicated by no change in surface IL-4Rα levels (Figure **S1G**). Furthermore, Gab1 recruitment to the IL-4R is reported to be essential for downstream Akt signal activation following IL-4 induction 37. Our results demonstrated that IL-4-induced association between IL-4Rα and Gab1was largely inhibited in Gαi1/3 DKO MEFs (Figure **S1H**), whereas IL-4Rα-JAK1 association, essential for STAT6 activation, remained intact (Figure **S1H**).

In further studies, we found that Gαi1/3 DKO blocked IL-4-induced Gab1 activation (Tyr-627 phosphorylation, Figure **S1I**). These results implied that Gαi1/3 are essential for IL-4-induced Gab1 activation, a necessary step for downstream Akt activation 37. In Gab1 KO MEFs (see our previous studies 15, 16, 18, 19), IL-4-induced phosphorylation of Akt, S6K and Erk1/2 was significantly reduced (Figure **S1J**). Thus, in MEFs Gαi1 and Gαi3 are required for IL-4Rα endocytosis as well as the recruitment and activation of the adaptor protein Gab1 in response to IL-4.

### Gαi1/3 knockdown inhibits IL-4-induced Akt activation and M2 polarization in BMDMs

To investigate the role of Gαi1/3 in IL-4-induced signaling in macrophages, we stimulated primary murine bone marrow-derived macrophages (BMDMs) with IL-4. Co-IP results demonstrated that IL-4Rα immunoprecipitated with Gαi1, Gαi3 and Gab1 (Figure **1A**). Knock-down of Gαi1/3 in BMDMs using Gαi1-shRNA lentivirus and/or Gαi3-shRNA lentivirus showed that while single knockdown of Gαi1 or Gαi3 partially inhibited Akt activation, Gαi1/3 double knockdown inhibited Akt to a greater extent (Figure **1B**). These results indicate that Gαi1 and Gαi3 are required for IL-4-induced Akt activation in BMDMs.

Previous studies have shown that IL-4 activates Akt and promotes M2 polarization of macrophages 14. In contrast, inhibition of Akt hinders IL-4-induced expression of M2 polarization markers, including *Arg1*, *Fizz1*, *Mgl2*, and *Mgl1* 14. As Gαi1/Gαi3 silencing inhibited IL-4-induced Akt activation, we hypothesized that Gαi1 and Gαi3 is necessary for IL-4-induced M2 polarization. Examining M2 marker expression by qPCR, we found that IL-4 significantly increased mRNA expression of *Arg1*, *Fizz1*, *Mgl1*, and *Mgl2* in control BMDMs (Figure **1C**-**F**). Significantly, shRNA-mediated knockdown of Gαi1 or Gαi3 potently inhibited mRNA levels of M2 markers (Figure **1C**-**F**). The combined knockdown of Gαi1 and Gαi3 resulted in further suppression of IL-4-induced M2 marker expression (Figure **1C**-**F**). In BMDMs IL-4-induced Arg1 and Fizz1 protein expression was also inhibited with Gαi1 or Gαi3 single knockdown (Figure **1G**), being more significant with Gαi1 and Gαi3 double knockdown (“sh-Gαi1/3”, Figure **1G**).

We also assessed arginase-1 activity by measuring urea production, and found that IL-4 stimulation increased activity in BMDMs (Figure **1H**). This effect was attenuated by Gαi1/3 shRNA (Figure **1H**). Interestingly, the deficits in M2 polarization appear to be selective, as induction of PPAR-γ by IL-4 was unaffected in Gαi1/3-shRNA BMDMs (Figure **1I**). Viability of BMDMs, tested by CCK-8 optical density (OD), was not affected by Gαi1/3 shRNA (Figure **1J**).

### Gαi1/3 association with APPL1 mediates IL-4Rα internalization and endosomal traffic, essential for IL-4-induced Gab1-Akt-mTOR signalling and M2 responses in macrophages

**Figure 2:**
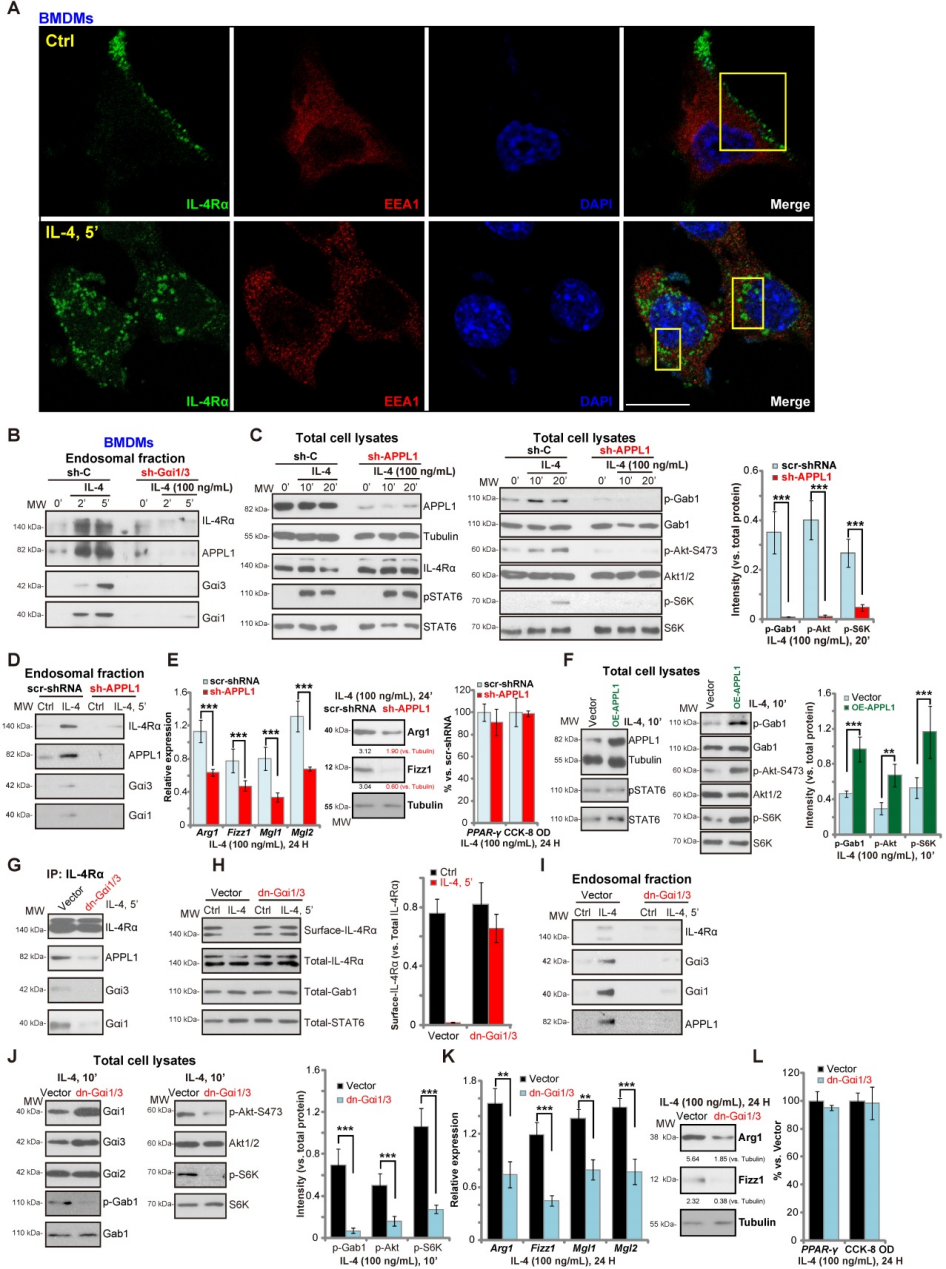
** Gαi1/3 association with APPL1 mediates IL-4Rα internalization and endosomal traffic, essential for IL-4-induced Gab1-Akt-mTOR signaling transduction and M2 responses in macrophages.** Primary cultured murine bone marrow-derived macrophages (BMDMs) were treated with IL-4 (100 ng/mL) for 5 min, confocal images were taken to demonstrate the locations of IL-4Rα, EEA1 (the early endosome marker) and DAPI (the nuclear marker) (**A**); Stable BMDMs, expressing the scramble control shRNA (“sh-C”), Gαi1 shRNA and Gαi3 shRNA (“sh-Gαi1/3”), were treated with or without IL-4 (100 ng/mL) for applied time periods, and tested by Western blotting of listed proteins in total endosomal fractions (**B**). Stable BMDMs, expressing sh-C or APPL1 shRNA (“sh-APPL1”), were treated with or without IL-4 (100 ng/mL) for listed time periods, listed proteins in total cell lysates (**C**) and endosomal fractions (**D**) were tested. Twenty-four hours after IL-4 treatment, relative expression of listed genes (mRNAs and proteins) was shown (**E**); The cell viability was tested as well (**E**). Stable BMDMs, with the lentiviral APPL1 construct (“OE-APPL1”) or empty vector (“Vector”), were treated with IL-4 (100 ng/mL) for 10 min, and were tested by Western blotting of listed proteins (**F**); BMDMs with the empty vector (“Vector”) or the dominant negative Gαi1 construct plus dominant negative Gαi3 construct (“dn-Gαi1/3”), were treated with IL-4 (100 ng/mL), IL-4Rα-APPL1-Gαi1/3 association was tested by co-immunoprecipitation (“IP: IL-4Rα”) (**G**); Expression of listed proteins, in total cell lysates (“Total”), plasma surface (“Surface”) and endosomal fractions, were tested by Western blotting assays (**H-J**). Twenty-four hours after IL-4 treatment, relative expression of listed genes (mRNAs and proteins) was shown (**K**), with cell viability tested as well (**L**). ***P < 0.001, **P < 0.01.Scale bar=25 µm (**A**).

We have previously shown that Gαi1/3 association with receptor tyrosine kinases (RTKs) are essential for their endocytosis and endosomal traffic 15, 16. Confocal studies in BMDMs demonstrated that following IL-4 stimulation, membrane IL-4R**α** translocated to signaling endosomes, colocalizing with the early endosomal marker EAA1 (Figure **2A**). APPL1 (adaptor containing a pleckstrin homology (PH) domain, phosphotyrosine-binding (PTB) domain, and leucine zipper motif 1) is required for endocytosis and signal transduction in signaling endosomes 38, 39. By isolating endosomal fractions 25, we showed that IL-4Rα, APPL1, Gαi1 and Gαi3 were enriched in IL-4-stimulated BMDMs, but not following Gαi1/3 silencing (Figure **2B**). Thus, Gαi1/3 are essential for IL-4Rα-APPL1 endosomal trafficking in IL-4-stimulated BMDMs.

Importantly, shRNA-mediated knockdown of APPL1 attenuated IL-4-induced phosphorylation of Gab1, Akt and S6K1 (Figure **2C**), similarly to the effect of Gαi1/3 double shRNA (Gαi1/3 DshRNA). Again, IL-4-induced STAT6 phosphorylation was not significantly affected (Figure **2C**). APPL1 shRNA potently inhibited IL-4-induced Gαi1, Gαi3 and APPL1 endosomal trafficking (Figure **2D**). Functional studies showed that IL-4-induced expression of M2 markers, *Arg1*, *Fizz1*, *Mgl1*, and *Mgl2*, were also significantly reduced in APPL1-silenced BMDMs (Figure **2E**), whereas the induction of *PPAR-γ* and cell viability was unchanged by APPL1 shRNA (Figure **2E**). Conversely, ectopic overexpression of APPL1 (“OE-APPL1”) augmented IL-4-induced Gab1, Akt, and S6K1 phosphorylation without affecting STAT6 phosphorylation (Figure **2F**). These results show that APPL1 is a key adaptor protein for IL-4-induced IL-4Rα endosomal trafficking and signaling.

The results were further validated using dominant negative (dn) mutants of Gαi1 and Gαi3 that precluded Gαi1/3 binding to adaptor/associated proteins 18, 19 leading to defective M2 responses in BMDMs. As shown dn-Gαi1/3 attenuated IL-4-induced IL-4Rα-APPL1-Gαi1/3 association in BMDMs (Figure **2G**). Furthermore, IL-4Rα internalization (Figure **2H**), endosomal translocation (Figure **2I**), and Akt-S6K activation were potently inhibited (Figure **2J**). Expression of the M2 markers, *Arg1*, *Fizz1*, *Mgl1*, and *Mgl2*, in IL-4-treated BMDMs was also significantly inhibited by dn-Gαi1/3 (Figure **2K**). IL-4-induced *PPAR-γ* expression and viability of BMDMs was unaffected (Figure **2L**). These results suggest that in response to IL-4 stimulation, Gαi1 and Gαi3 associates with IL-4Rα and APPL1, resulting in IL-4R internalization and endosomal trafficking, which is essential for Gab1-Akt-mTOR signal transduction and M2 responses in BMDMs.

### Gαi1/3 bind to the intracellular domain of IL-4Rα

**Figure 3:**
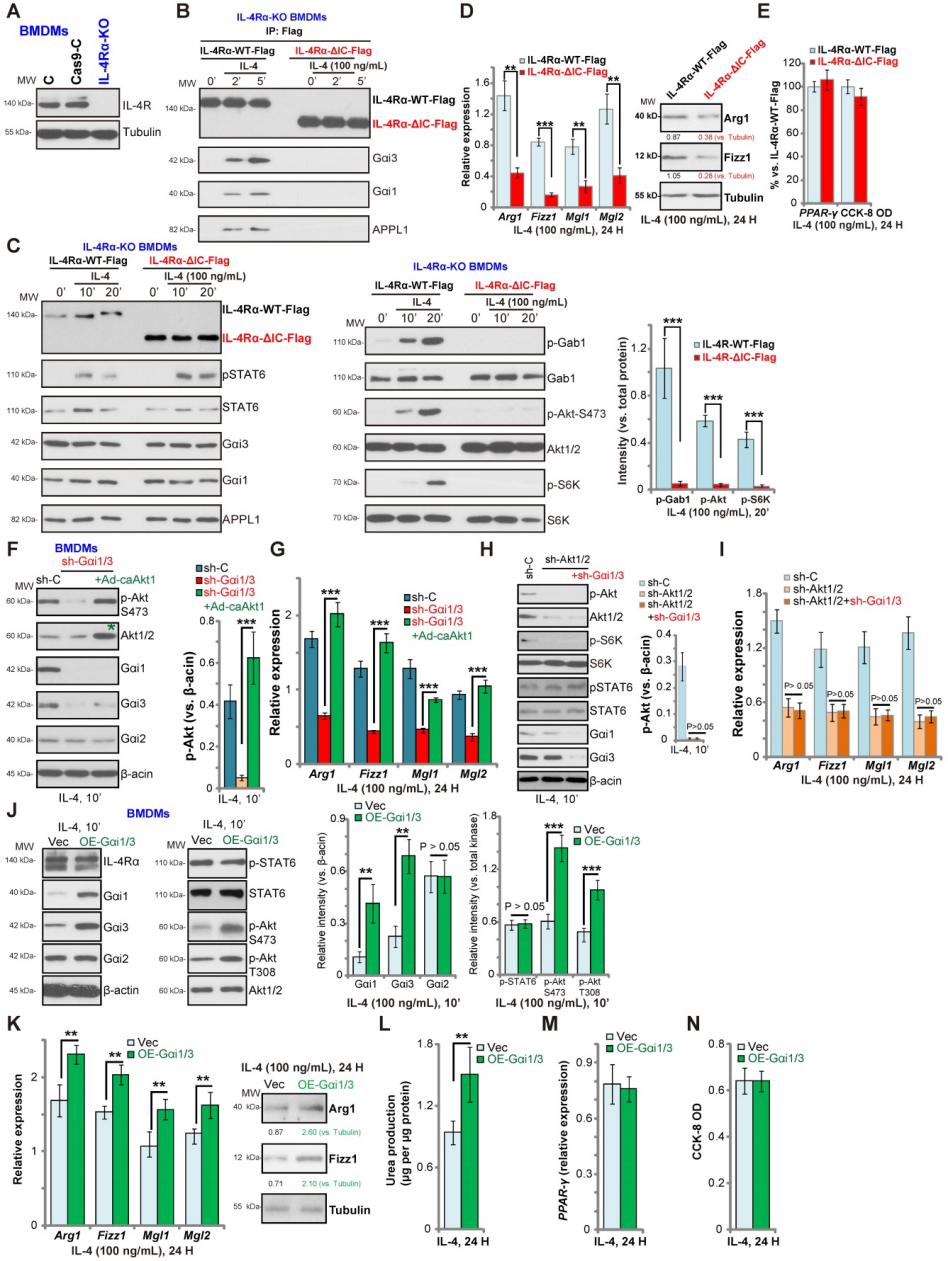
** Gαi1/3 bind to the intracellular domain of IL-4Rα.** Expression of IL-4Rα and Tubulin in the parental control BMDMs (“C”), the stable BMDMs with CRISPR-Cas9-IL-4Rα-KO construct (“IL-4Rα-KO”) or control vector (“Cas9-C”) was shown (**A**). The IL-4Rα-KO BMDMs were further infected with lentiviral wild-type IL-4Rα construct (Flag-tagged, “IL-4Rα-WT-Flag”) or the lentiviral intracellular domain-depleted IL-4Rα construct (Flag-tagged, “IL-4Rα-ΔIC-Flag”), subjected to puromycin selection to establish stable BMDMs; Established BMDMs were further treated with IL-4 (100 ng/mL) for applied time, and tested by Co-IP (“IP: Flag”) to examine IL-4Rα-APPL1-Gαi1/3 association (**B**), with expression of listed proteins in total cell lysates tested by Western blotting assays (**C**). Twenty-four hours after IL-4 treatment, relative expression of listed genes (mRNAs and proteins) was shown (**D** and **E**), with the cell viability tested as well (**E**). The scramble control shRNA (“sh-C”)-expressing BMDMs or Gαi1 shRNA plus Gαi3 shRNA (“sh-Gαi1/3”)-expressing BMDMs (with or without the constitutively-active Akt1 adenovirus [“+Ad-caAkt1”, with green star marker]) were treated with IL-4 (100 ng/mL) for 10 min, tested by immunoblotting of listed proteins (**F**, Akt phosphorylation was quantified); Relative expression of listed genes (24h after IL-4 treatment) was shown (**G**); The sh-C BMDMs or Akt1/2-shRNA-expressing stable BMDMs (with or without sh-Gαi1/3) were treated with IL-4 (100 ng/mL) for 10 min, tested by immunoblotting of listed proteins (**H**, Akt phosphorylation was quantified); Relative expression of listed genes (24h after IL-4 treatment) was shown (**I**); Stable BMDMs, with the adenovirus Gαi1 construct plus the adenovirus Gαi3 construct (“OE-Gαi1/3”) or empty vector (“Vec”), were treated with IL-4 (100 ng/mL) for applied time, and were tested by Western blotting of listed proteins (**J**, results were quantified); Relative expression of listed genes (mRNAs and proteins) was tested by qPCR assays (**K** and **M**); The urea production was also tested (**L**), with cell viability tested by CCK-8 (**N**).“Ctrl” stands for untreated control BMDMs. ***P < 0.001, **P < 0.01.

IL-4-induced IL-4Rα endocytosis is dependent on its intracellular domain (IC domain) 26, 27. However, IL-4Rα endocytosis is not functionally required for the JAK-STAT6 signal transduction 26, 27. We hypothesized that Gαi1/3 bind to the IC domain of IL-4Rα to mediate endocytosis, endosomal trafficking, and downstream Gab1-Akt-mTOR signaling activation. To test this hypothesis, we expressed normal and IC domain-deleted IL-4Rα into the CRISPR-Cas9-IL-4Rα-KO BMDMs (“IL-4Rα-KO BMDMs”, Figure **3A**). The lentiviral wild-type IL-4Rα (Flag-tagged, “IL-4Rα-WT-Flag”) construct or the lentiviral IC domain-depleted IL-4Rα (Flag-tagged, “IL-4Rα-ΔIC-Flag”) was transduced into the IL-4Rα-KO BMDMs (Figure **3B**). Co-IP results demonstrated that in response to IL-4 stimulation, Gαi1/3 and APPL1 associated with the IL-4Rα-WT-Flag, but not with the IL-4Rα-ΔIC-Flag in BMDMs (Figure **3B**). Furthermore, IL-4-induced phosphorylation of Gab1, Akt and S6K1 was blocked in BMDMs transduced with IL-4Rα-ΔIC-Flag, while expression of Gαi1/3, APPL1 and STAT6, as well as IL-4-induced STAT6 phosphorylation were intact (Figure **3C**). These results suggest that Gαi1/3 binds to the IC domain of IL-4Rα, required for IL-4Rα endosomal trafficking and Gab1-Akt-mTOR activation. Functionally, IL-4Rα-ΔIC-Flag transduction resulted in robust inhibition of IL-4-induced expression of M2 markers, *Arg1*, *Fizz1*, *Mgl1*, and *Mgl2* in BMDMs (Figure **3D**). IL-4-induced Arg1 and Fizz1 protein expression was largely inhibited as well (Figure **3D**). Again, IL-4-induced *PPAR-γ* expression as well as BMDM viability were unchanged (Figure **3E**).

Akt signaling has been shown to play a key role in M2 activation in BMDMs 14, 37, 40. To determine whether Akt re-activation is sufficient to rescue M2 polarization, we expressed a constitutively active Akt1 (caAkt1) adenovirus 21, which restored Akt activity in Gαi1/3 DshRNA-expressing BMDMs (Figure **3F**). Significantly, in the Gαi1/3-silenced BMDMs, Ad-caAkt1 restored *Arg1*, *Fizz1*, *Mgl1*, and *Mgl2* expression in response to IL-4 (Figure **3G**). Further studies demonstrated that shRNA-mediated silencing of Akt1/2 inhibited IL-4-induced Akt-S6K phosphorylation as well as *Arg1*, *Fizz1*, *Mgl1* and *Mgl2* expression in BMDMs (Figure **3H**). Significantly, Gαi1/3 DshRNA failed to further inhibit expression of M2 markers in Akt1/2-silenced BMDMs (Figure **3I**). IL-4-induced STAT6 phosphorylation was again not affected by Akt1/2 shRNA, or plus Gαi1/3 DshRNA (Figure **3H**).

### Gαi1 and Gαi3 overexpression promotes IL-4-induced Akt activation and M2 polarization in BMDMs

Based on our results, we hypothesized that Gαi1 and Gαi3 overexpression would promote IL-4-induced M2 polarization. To test this, stable Gαi1 and Gαi3 overexpressing BMDMs were established (“OE-Gαi1/3”, Figure **3J**). In OE-Gαi1/3 BMDMs, IL-4-induced Akt phosphorylation was significantly increased (Figure **3J**). Expression of IL-4Rα, Gαi2 and STAT6, as well as IL-4-induced STAT6 phosphorylation, were unchanged between control BMDMs and OE-Gαi1/3 BMDMs (Figure **3J**). IL-4-induced *Arg1*, *Fizz1*, *Mgl1* and *Mgl2* expression (Figure **3K**) and urea production (Figure **3L**) were further increased in OE-Gαi1/3 BMDMs. OE-Gαi1/3 also augmented IL-4-induced Arg1 and Fizz1 protein expression (Figure **3K**). Induction of *PPAR-γ* by IL-4 was not affected by Gαi1/3 overexpression (Figure **3M**). The cell viability was unchanged between control and OE-Gαi1/3 BMDMs (Figure **3N**). Thus, Gαi1/3 overexpression promoted IL-4-induced Akt activation and M2 polarization in BMDMs.

In primary cultured alveolar macrophages (AMs), lentiviral shRNAs were applied to knockdown Gαi1 and Gαi3 (sh-Gαi1/3, Figure **S2A**). IL-4-induced phosphorylation of Akt and S6K1 was almost completely blocked by sh-Gαi1/3 in AMs (Figure **S2A**). Expression of IL-4Rα and Gαi2 were however unchanged (Figure **S2A**). Importantly, IL-4-induced expression of M2 polarization markers, including*Arg1*, *Fizz1*, *Mgl2*, and *Mgl1*, was ameliorated by sh-Gαi1/3 in AMs (Figure **S2B**). On the contrary, stable transduction of Ad-Gαi1 plus Ad-Gαi3 increased Gαi1/3 expression (“OE-Gαi1/3”) in primary AMs (Figure **S2C**). Akt and S6K1phosphorylation in response to IL4 was intensified in OE-Gαi1/3 AMs (Figure **S2C**). *Arg1*, *Fizz1*, *Mgl2*, and *Mgl1*mRNA expression was enhanced as well (Figure **S2D**).

### Gαi1 and Gαi3 DKO inhibits IL-4-induced Akt activation and M2 polarization in BMDMs

**Figure 4:**
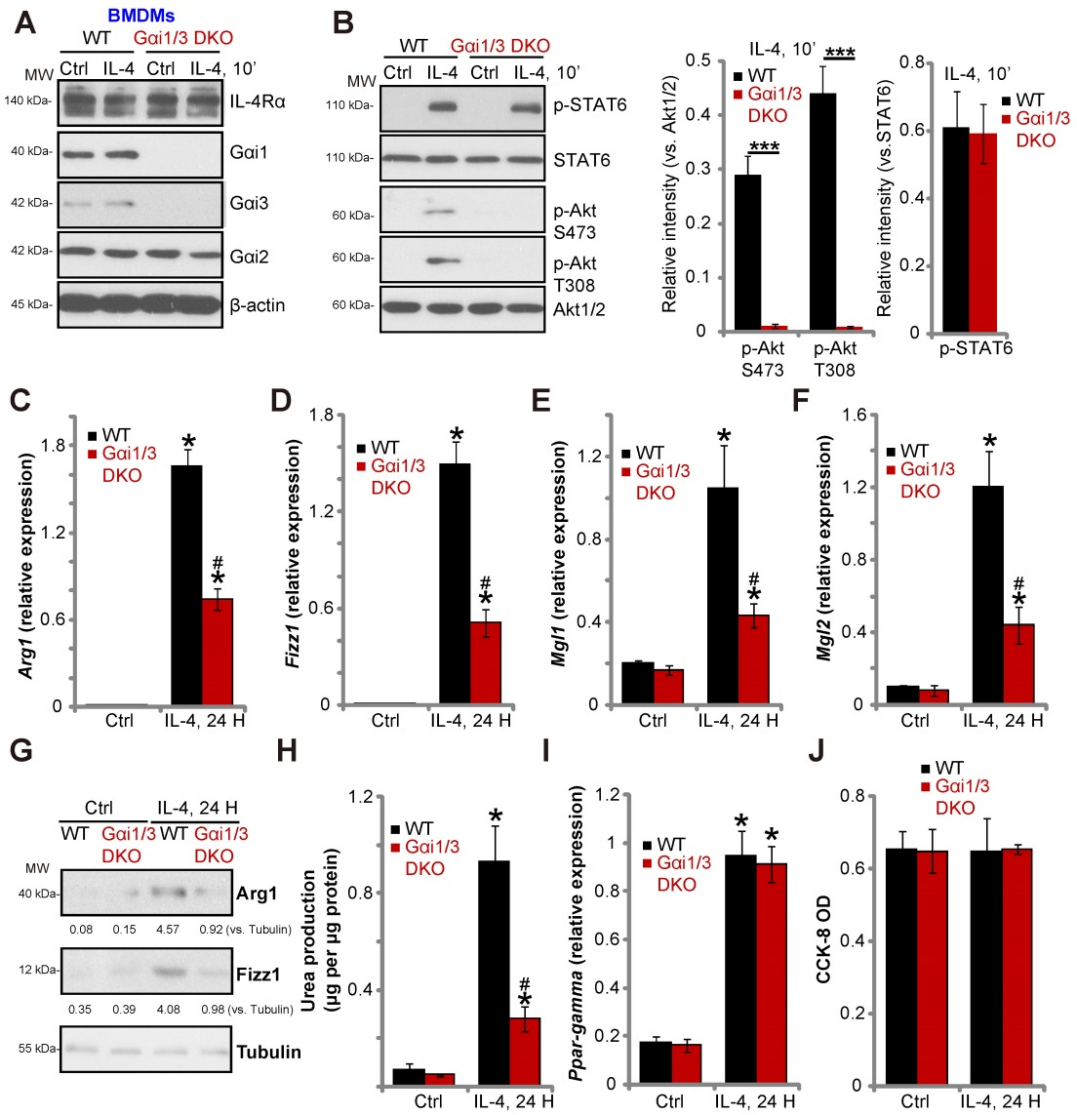
** Gαi1 and Gαi3 DKO inhibits IL-4-induced Akt activation and M2 polarization in BMDMs.** BMDMs, derived from both WT mice and Gαi1 and Gαi3 DKO mice (five week old), were treated with IL-4 (100 ng/mL) for applied time, and were tested by Western blotting of listed proteins (**A** and **B**); Expression of listed genes (mRNAs and proteins) was tested by qPCR (**C**-**G**, **I**); The urea production was also tested (**H**), with cell viability tested byCCK-8 assay (**J**). “Ctrl” stands for untreated control BMDMs. *P < 0.001 (**B**). *P < 0.001 vs. “Ctrl” treatment in “WT” DMEMs (**C**-**I**).**^ #^*P*** < 0.001 vs. IL-4 treatment in “WT” DMEMs (**C**-**H**).

To confirm the essential role of Gαi1/3 in M2 polarization, we compared IL-4-induced activity between BMDMs derived from WT mice and Gαi1/3 DKO mice 15, 16. Gαi1 and Gαi3 were depleted in BMDMs from Gαi1/3 DKO mice, while Gαi2 expression was intact (Figure **4A**). IL-4-induced Akt phosphorylation was blocked in Gαi1/3 DKO BMDMs, whereas IL-4Rα and STAT6 expression, as well as IL-4-induced STAT6 phosphorylation were unaffected (Figure **4A** and **B**). As compared to the WT BMDMs, IL-4-induced* Arg1*, *Fizz1*, *Mgl1*, and *Mgl2* expression (Figure **4C-G**) as well as urea production (Figure **4H**) were impaired in Gαi1/3 DKO BMDMs. *PPAR-γ* expression and cell viability was unaffected (Figure **4I** and **J**).

### Gαi1 and Gαi3 are required for IL-4-induced Akt activation and Th2 response in human macrophages

**Figure 5:**
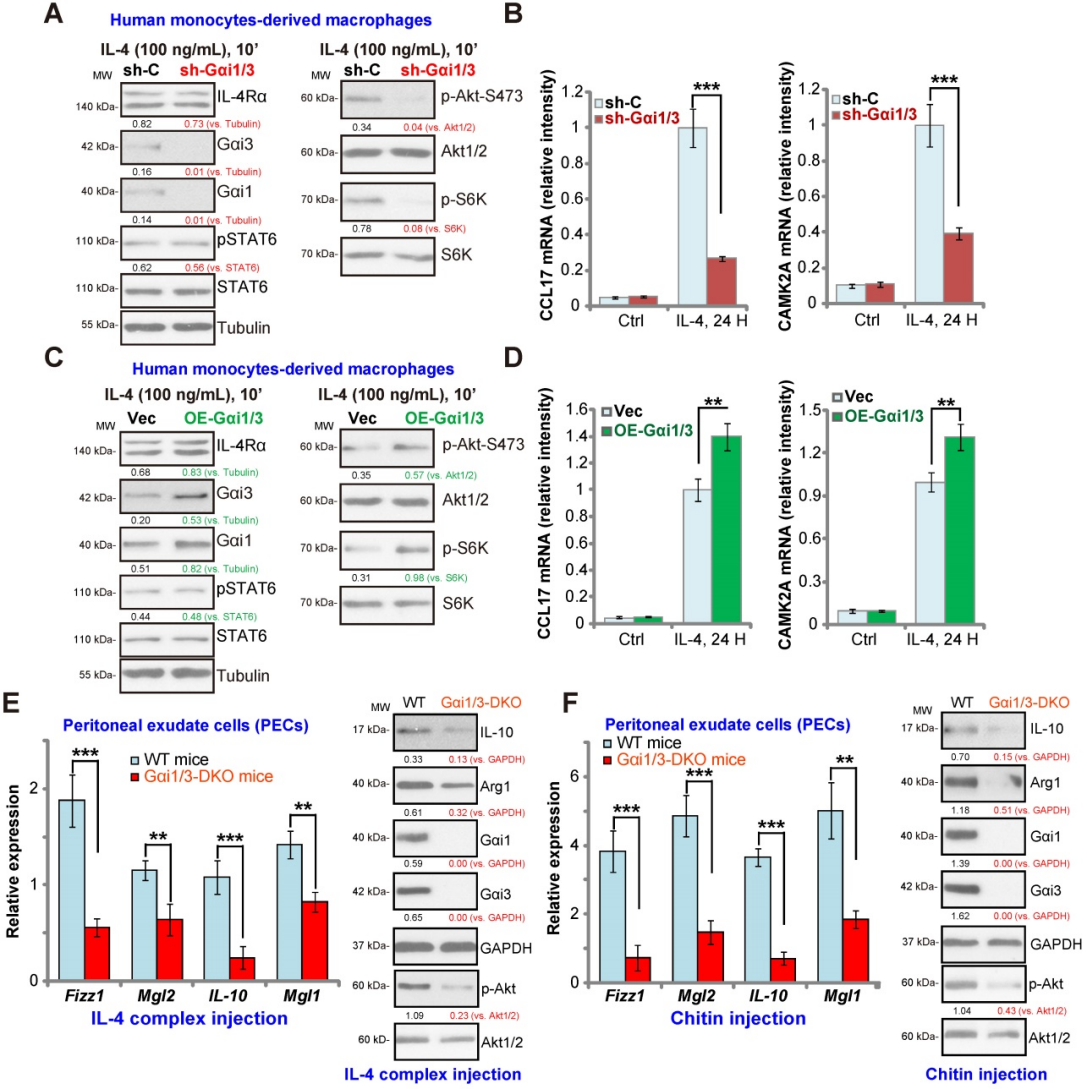
** Impaired M2 polarization in human macrophages and Gαi1/3 DKO mice.** Human monocytes-derived macrophages (MDMs) were transduced with the lentiviral scramble control shRNA (“sh-C”) or the lentiviral Gαi1 shRNA plus Gαi3 shRNA (“sh-Gαi1/3”) (**A** and **B**), the adenovirus Gαi1 construct plus the adenovirus Gαi3 construct (“OE-Gαi1/3”) or empty vector (“Vec”) (**C** and **D**), MDMs were then treated with or without IL-4 (100 ng/mL) for 10 min, and tested by Western blotting of listed proteins (**A** and **C**). Twenty-four hours after IL-4 treatment, relative expression of listed genes was shown (**B** and **D**); (**E**) M2 genes (*Fizz1*, *Mgl2*, *IL-10* and *Mgl1*) expression and Akt activation in peritoneal exudate cells (PECs) from WT and Gαi1/3 DKO mice four days post intraperitoneal (IP) injection with IL-4 complex on days 0 and 2. (F) Relative expression of M2 genes (*Fizz1*, *Mgl2*, *IL-10* and *Mgl1*, mRNAs and proteins) as well as Akt activation in PECs from WT and Gαi1/3 DKO mice 48h after IP injection with chitin. In each experiment, n=5 (five replicated wells/dishes). Experiments were repeated five times (five mice per group), data of all repeated experiments were pulled together to calculate mean ±SD. ***P < 0.001, **P < 0.01.

In human monocytes-derived macrophages (MDMs), shRNA (see our previous study 15) was utilized to knockdown both Gαi1 and Gαi3. The applied shRNA lentivirus resulted in significant Gαi1 and Gαi3 protein double downregulation (sh-Gαi1/3, Figure **5A**). In line with the results in BMDMs, IL-4-induced phosphorylation of Akt and S6K1 was largely inhibited by sh-Gαi1/3 in MDMs (Figure **5A**). In contrast, expression of IL-4Rα and STAT6 as well as IL-4-induced STAT6 phosphorylation were unchanged (Figure **5A**). As there is a significant difference between the transcriptional response toward IL-4 in human and murine macrophages 41, 42, we analyzed the potential role of sh-Gαi1/3 on expression of Th2 response genes in MDMs, including *CCL17* and *CAMK2A* 41, 42. The qPCR assay results, Figure **5B**, showed that IL-4 significantly increased expression of *CCL17* and *CAMK2A* in MDMs. This response was largely inhibited by sh-Gαi1/3 (Figure **5B**).

As Gαi1 and Gαi3 silencing inhibited IL-4-induced expression of Th2 response genes, we hypothesized that Gαi1 and Gαi3 overexpression would facilitate IL-4-induced Th2 response in human macrophages 43. To examine this, adenovirus Gαi1 (“Ad-Gαi1”, no Tag 15) and Gαi3 constructs (“Ad-Gαi3”, no Tag 15) were utilized to establish stable MDMs exogenously over-expressing Gαi1 and Gαi3, OE-Gαi1/3. As shown Gαi1 and Gαi3 protein were both elevated in OE-Gαi1/3 MDMs (Figure **5C**). As a result, IL-4-induced phosphorylation of Akt and S6K1 was significantly augmented (Figure **5C**). IL-4Rα and STAT6 expression as well as IL-4-induced STAT6 phosphorylation were unchanged (Figure **5C**). Importantly, IL-4-induced expression of Th2 response genes, *CCL17* and *CAMK2A*, was potentiated with Gαi1 plus Gαi3 overexpression (Figure **5D**), demonstrating that Gαi1/3 are important for IL-4-induced Akt signaling and Th2 response in human macrophages.

### Defective M2 polarization in Gαi1/3 DKO mice

To test whether Gαi1/3 deficiency could impair M2 polarization *in vivo* 14, an IL-4/anti-IL-4 complex was intraperitoneally injected to elicit IL-4-dependent M2 response in both WT mice and Gαi1/3 DKO mice 15, 16. Significantly, induction of M2 genes, including *Fizz1*, *Mgl2*, *IL-10* and *Mgl1* was inhibited in the peritoneal exudate cells (PECs) from Gαi1/3 DKO mice (Figure **5E**). IL-4 complex-induced Arg1 and IL-10 protein expression was inhibited as well (Figure **5E**). Akt phosphorylation was inhibited as well (Figure **5E**). To further support our hypothesis, the chitin administration model was applied, causing IL-4-dependent recruitment and polarization of M2 macrophages 14. As compared to the WT mice, in PECs from Gαi1/3 DKO mice, *Fizz1*, *Mgl2*, *IL-10* and *Mgl1* mRNA expression, Arg1 and IL-10 protein expression as well as Akt phosphorylation were significantly decreased in response to chitin (Figure **5F**). Thus, these results demonstrate that Gαi1/3 DKO impairs M2 polarization *in vivo*.

### Ovalbumin-induced airway inflammation and hyperresponsiveness are largely impaired in Gαi1/3 DKO mice

**Figure 6:**
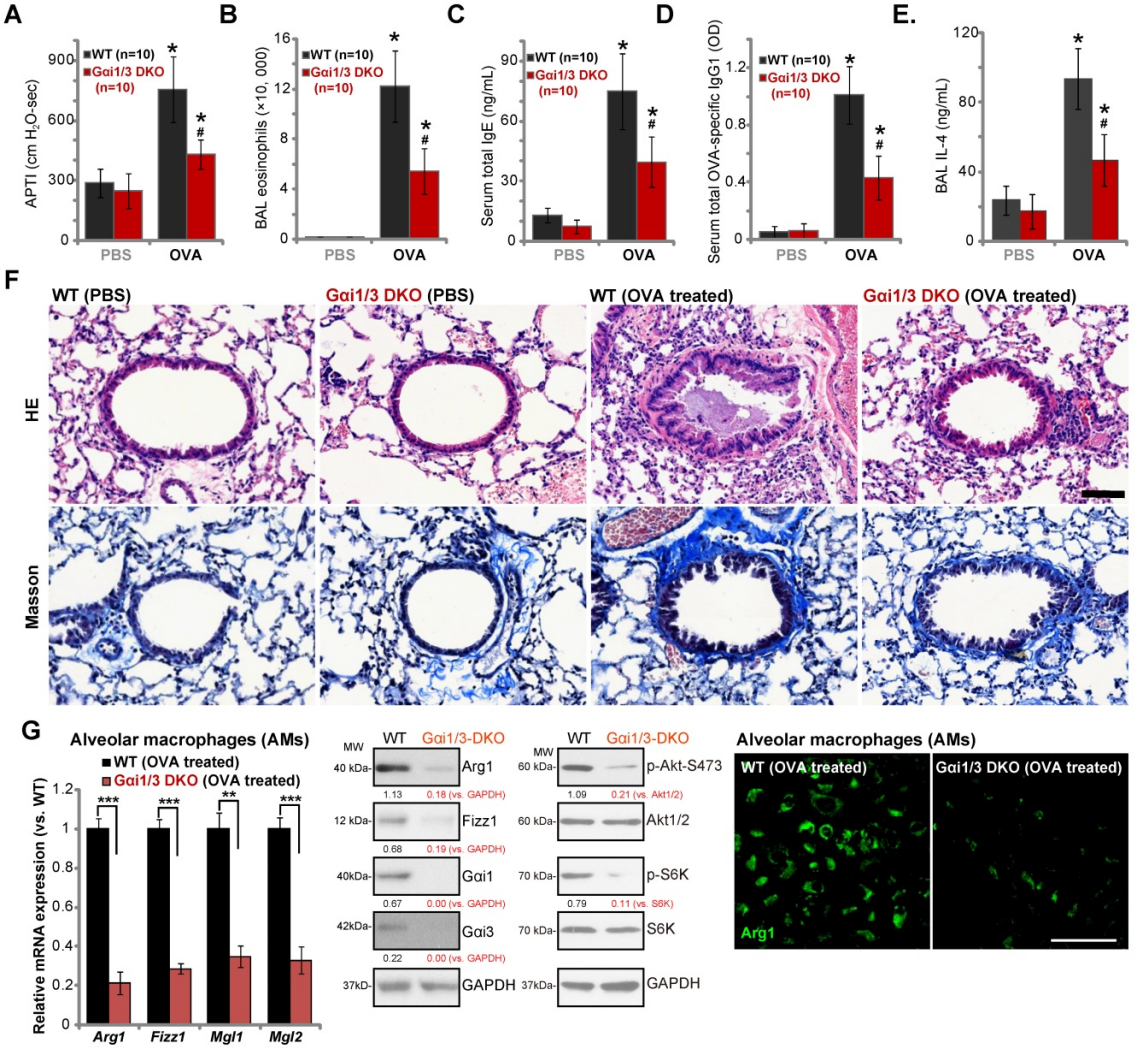
** Ovalbumin-induced airway inflammation and hyperresponsiveness are largely impaired in Gαi1/3 DKO mice.** WT or Gαi1/3 DKO mice (10 mice per group) were first sensitized and then challenged by OVA or PBS for three days. The airway responsiveness to intravenous acetylcholine chloride (Ach) administration was determined (**A**); The number of bronchoalveolar lavage (BAL) fluids eosinophils (**B**), serum total IgE contents (**C**), serum total OVA-specific IgG1 (**D**) and IL-4 contents in BAL fluids (**E**) were determined. Lungs were also fixed and subjected to HE staining and Masson staining (**F**). Scale Bar= 50 µm. Alveolar macrophages (AMs) were isolated and relative expression of listed genes was tested by qPCR, Western blotting and immunofluorescence assays (**G**). * P < 0.05 vs. PBS treatment in WT mice (**A**-**E**). **^#^*P*** < 0.05 *vs.* OVA treatment in WT mice (**A**-**E**). ***P < 0.001 (**G**), **P < 0.01 (**G**). Scale bar= 100 µm (**G**).

Lung IL-4 signaling and M2 macrophages are key regulators of airway responses to inhaled allergens, participating in poor lung function in allergic asthma 32, 44-46. We therefore compared the effects of OVA sensitization and challenge on the development of allergic airway responses in WT and Gαi1/3 DKO mice. Assessing airway responsiveness to intravenous acetylcholine chloride (Ach) administration 32, following OVA sensitization and challenge WT mice developed significant increases in airway pressure time index (APTI) after Ach administration (Figure **6A**). In contrast, the airway reactivity was significantly lower in OVA-treated Gαi1/3 DKO mice (***P*** < 0.05 vs. WT mice, Figure **6A**), suggesting that Gαi1/3 are involved in OVA-induced airway hyperresponsiveness (AHR). IL-4 plays an important role in eosinophilia by increasing IL-5 production and upregulation of endothelial VCAM-1 expression, to promote attachment and migration of eosinophils 44. As expected, OVA sensitization and challenge induced a striking increase in the number of eosinophils in bronchoalveolar lavage (BAL) fluids of WT mice (Figure **6B**). Although increases in BAL eosinophils were detected OVA-treated Gαi1/3 DKO mice, they were much lower than OVA-treated WT animals (Figure **6B**).

IL-4 is also important for IgE synthesis required for the pathogenesis of allergic responses. We found that OVA-treated WT mice produced a large amount of serum IgE (Figure **6C**). OVA-stimulated IgE production was however attenuated in Gαi1/3 DKO mice (***P*** < 0.05 vs. WT mice, Figure **6C**). In addition, we examined serum levels of OVA-specific IgG1, a method utilized to assess Th2 cytokines *in vivo*. Compared to OVA-treated WT mice (Figure **6D**), the serum OVA-specific IgG1 levels were inhibited in Gαi1/3 DKO mice (***P*** < 0.05 *vs.* WT mice, Figure **6D**). In OVA-challenged WT mice, IL-4 contents in BAL were significantly increased, but were much lower in the BAL of OVA-challenged DKO mice (see revised Figure **6E**). These results further demonstrate inhibition of the Th2 response in OVA-treated Gαi1/3 DKO mice.

Examining pulmonary histopathology with HE and Masson staining demonstrated that in OVA-treated WT mice the bronchial wall was thickened and the lumen was narrow (Figure **6F**). A significant amount of mucus was detected in the lumen, with several red mucus plugs observed as well (Figure **6F**). In addition, a large number of inflammatory cells, including lymphocytes, eosinophils, neutrophils, were infiltrated into bronchus and blood vessels (Figure **6F**). In contrast, in the lung of OVA-treated Gαi1/3DKO mice, the bronchioles and alveoli were mainly intact, with few necrotic or exfoliated epithelial cells (Figure **6F**). The number of infiltrated inflammatory cells was significantly lower when compared to WT mice (Figure **6F**). These results show that Gαi1/3DKO protects against OVA-induced airway hyperresponsiveness and mucus production. Alveolar macrophages (AMs) were isolated from OVA-treated mice. In AMs of OVA-treated Gαi1/3 DKO mice expression of M2 genes (*Arg1*, *Fizz1*, *Mgl2*, and *Mgl1*) was significantly lower than that in AMs of OVA-treated WT mice (Figure **6G**). Western blotting and immunofluorescence assay results further confirmed Arg1 and Fizz1 protein levels in AMs of OVA-treated Gαi1/3 DKO mice were significantly lower than those in AMs of OVA-treated WT mice (Figure **6G**). Akt-S6K phosphorylation was inhibited as well (Figure **6G**). These results further suggest that Gαi1/3 are important genes for IL4-induced macrophage M2 polarization and Th2 response *in vivo*.

## Discussion

It is widely accepted that IL-4 induced M2 polarization occurs through IL-4Rα to recruit IL-2Rγ (type I receptor), to activate the non-receptor tyrosine kinase JAK1, leading to phosphorylation, dimerization and activation of STAT6 47-50. Recent studies, however, have proposed that IL-4Rα-activated PI3K-Akt signaling, in parallel to the JAK1-STAT6 cascade, also plays a key role in regulating and maintaining M2 responses 14, 37, 40. Pharmacological inhibition of Akt significantly inhibits M2 activation in BMDMs, while constitutively-active Akt (caAkt) enhances M2 activation in BMDMs 14, 37, 40. However, the underlying mechanisms mediating IL-4-induced M2 activation through Akt remain largely elusive.

The present study provides a novel mechanism for the role of Gαi1/3 in IL-4 signal transduction underlying M2 responses. We show that Gαi1/3 are required for IL-4-induced Akt activation. In BMDMs, human macrophages and MEFs, Gαi1/3 KO/shRNA, CRISPR-induced Gαi1/3 gene editing, or dominant negative Gαi1/3 mutations potently inhibited IL-4-induced Akt activation. Conversely, overexpression of Gαi1/3 facilitated Akt activation by IL-4. Importantly, IL-4-induced STAT6 activation was intact regardless of Gαi1/3 status. Functional studies demonstrated that BMDMs or human macrophages with Gαi1/3 deficiency were resistant to IL-4-induced M2 polarization, whereas Gαi1/3-overexpressing BMDMs or human macrophages displayed preferred M2 responses to IL-4. *In vivo* M2 activation, induced by injection of IL-4/anti-IL-4 complex or chitin, was significantly inhibited in Gαi1/3 DKO mice. Our findings support that Gαi1/3 are required for IL-4-induced Akt activation and M2 polarization.

Following IL-4 stimulation, IL-4Rα is internalized to mediate ligand uptake 26. The intracellular domain of IL-4Rα initiates Rac1-, Pak- and actin-mediated endocytosis, leading to an increased receptor density at endosomes 36. Whether IL-4Rα endocytosis is essential for downstream signaling transduction is still under debate, and could be cell-type-dependent. Kurgonaite *et al.,* showed that in HEK293T cells IL-4Rα endocytosis is required for IL-4-induced JAK/STAT6 activation 36, although Friedrich *et al.,* demonstrated that IL-4Rα endocytosis is not functionally connected to JAK/STAT6 activation in macrophages 26.

Our previous studies show that Gαi1/3 play an essential role in the formation of the VEGFR2 endocytosis complex, required for VEGFR2 endocytosis and downstream signaling activation by VEGF 15. Furthermore, BDNF-induced TrkB endocytosis and downstream signaling activation are blocked in Gαi1/3-depleted cells and neurons 16. The results of the present study demonstrate that Gαi1 and Gαi3 physically associate with the intracellular domain of IL-4Rα, which is essential for IL-4Rα endocytosis and Akt activation, but not STAT6 activation. Disruption of IL-4Rα-Gαi1/3 association, by Gαi1/3 silencing/KO, dominant negative mutants, or though deletion of the intracellular domain of IL-4Rα, abrogated IL-4-induced IL-4Rα endocytosis, endosomal traffic, and Akt activation, while leaving STAT6 unaffected. Therefore, in IL-4-treated BMDMs, Gαi1/3-mediated IL-4Rα endocytosis and endosomal traffic are essential for activation of Akt, but not STAT6.

The APPL1 adaptor localized to endosomes serves as a platform for the assembly and trafficking of receptors and endosomal signaling 38, 39. Following IL-4 stimulation, we found that APPL1 association with Gαi1/3 and IL-4Rα is required for IL-4Rα endosomal traffic and Gab1-Akt-mTOR activation in BMDMs. IL-4-induced IL-4Rα endosomal traffic, Gab1-Akt-mTOR activation and M2 response were potently inhibited by APPL1 silencing. Conversely, ectopic overexpression of APPL1 promoted IL-4 signaling. Therefore, we propose that APPL1 is a critical adaptor protein for IL-4-induced M2 signaling.

Our previous studies have confirmed that in RTK signaling, Gαi1/3 are essential for Gab1 activation in response to various growth factors 17-19. In response to IL-4, macrophages can polarize towards M2, leading to expression of multiple M2 biomarkers, including *Arg1*, *Mgl1*, *Mgl2* and *Fizz1*. IL-4-induced activation of PI3K-AKT activation is vital in regulating expression of M2 markers 37. Following IL-4 stimulation, Gab1 preferentially interacted with p85 to activate PI3K-AKT signaling 37. In the present study, we show that Gab1 recruitment to IL4-activated IL-4Rα and subsequent activation were largely impaired when Gαi1/3 were silenced, depleted, or mutated. In Gab1 KO MEFs treated with IL-4, Akt activation was completely blocked, while Gαi1/3 expression and STAT6 activation were intact. Therefore, Gαi1/3 are required for IL4-induced Gab1 recruitment and activation, which mediates downstream Akt activation and M2 responses. One possibility is that IL-4-induced IL-4Rα endosomal traffic was disrupted with Gαi1/3 silencing, KO or mutation, that should block the docking of the adaptor protein Gab1, thus inhibiting Gab1 activation and downstream p85-Akt activation.

Our model is that Gαi1/3 are key signaling proteins for both LPS-induced M1 polarization and IL-4-induced M2 polarization, depending on the stimuli. Gαi1/3 are required for LPS-induced Gab1 activation via association with CD14 (see our previous study 51), explaining Gαi1/3's function in LPS-induced macrophage M1 polarization 51. Following IL-4 stimulation of STAT6 macrophages only polarize to M2 52. Significantly, we found that Gαi1/3 depletion did not affect IL-4-induced STAT6 activation. Therefore, Gαi1/3 depletion did not reverse M2 polarization towards M1, but rather only partially inhibited IL-4-induced M2 polarization by blocking Gab1-Akt activation. Indeed, Fan *et al.,* found that although LPS-induced production of M1 response genes, including tumor necrosis factor-α (TNF-α), interleukin-6 (IL-6) and IL-1β, were largely inhibited in Gαi1/3 DKO mice 53, 54 (also see our previous study 51). The production of M2 response gene, IL-10, was inhibited as well in Gαi1/3 DKO mice 53, 54. The role of Gαi proteins in macrophage polarization may warrant further investigation.

IL-4 and other Th2 cytokines are responsible for recruiting leukocytes to the site of inflammation, essential for IgE synthesis, airway eosinophilia, mucus secretion, and ultimately airway hyperresponsiveness (AHR) 55, 56. *Studies* have shown that injection or overexpression of IL-4 in the airways could induce airway eosinophilia and AHR 55. Here, using an asthma mouse model we found that OVA-stimulated IgE production, airway eosinophilia, inflammatory cells infiltration and AHR were largely impaired in Gαi1/3 DKO mice. Furthermore, expression of M2 markers in *ex vivo* alveolar macrophages-derived from Gαi1/3 DKO mice was significantly lower than that in alveolar macrophages of WT mice. The results of this study suggest that Gαi1/3 could be novel and key mediators of allergic asthma pathogenesis. Targeting Gαi1/3 could provide a new therapeutic modality for allergic asthma patients.

## Conclusion

The results of the present study reveal novel and essential roles of Gαi1/3 proteins in the control of IL-4 signaling, macrophage functions and M2 polarization, with broad implications for regulation of Th2 immunity, inflammation, and allergy.

## Supplementary Material

Supplementary figures.
