# Supplementary material for: Requirement of Gαi1 and Gαi3 in interleukin-4-induced signaling, macrophage M2 polarization and allergic asthma response
Source: Theranostics. 2021 Mar 4;11(10):4894–909. doi: 10.7150/thno.56383 (PMC7978294; doi:10.7150/thno.56383)
Supplement: Supplementary file 1 — Supplementary figures. [file thnov11p4894s1.pdf]

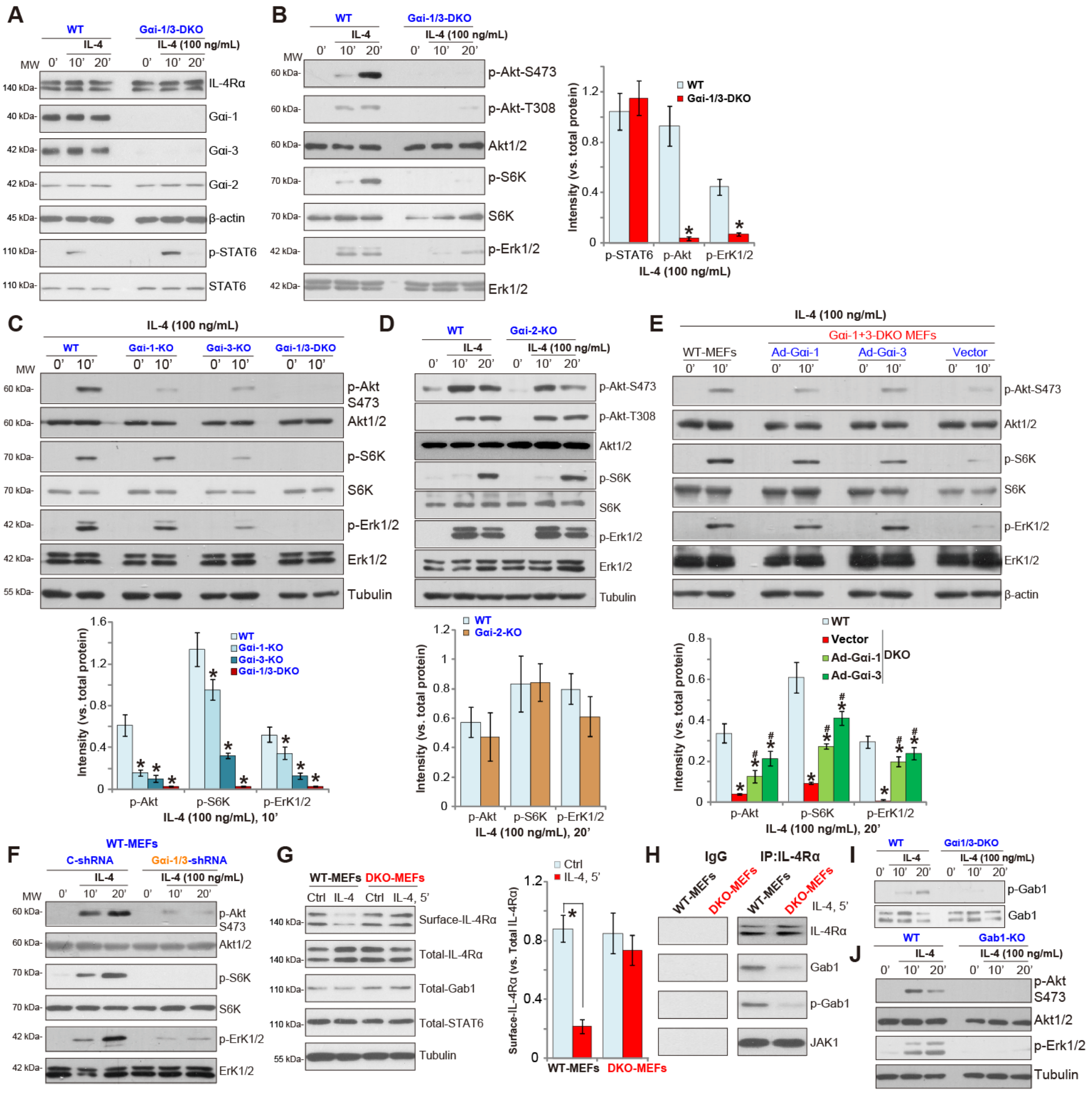

**Figure S1.** Wild-type (WT), Gαi1 and Gαi3 double knockout (DKO) (**A**-**B** and **I**), Gαi1, Gαi2 or Gαi3 single knockout (SKO) (**C** and **D**) mouse embryonic fibroblasts (MEFs) were treated with IL-4 (100 ng/mL) for applied time, tested by Western blotting of listed proteins (**A**-**D**, **I**); DKO MEFs were transiently transfected with the adenovirus Gαi1 construct ("Ad-Gαi1"), the adenovirus Gαi3 construct ("Ad-Gαi3") or the empty vector ("Vec"), IL-4-induced signaling was tested similarly (**E**). Stable WT MEFs with the scramble control shRNA ("C-shRNA"), Gαi1 shRNA and Gαi3 shRNA ("Gαi-1/3-shRNA"), were treated with IL-4 (100 ng/mL) for applied time, and were tested by Western blotting of listed proteins (**F**). WT and Gαi1/3 DKO MEFs were treated with or without IL-4 (100 ng/mL) for 5 min, expression of listed proteins in plasma membrane fractions and in total cell lysates was shown (**G**). WT and Gαi1/3 DKO MEFs were treated with IL-4 (100 ng/mL) for 5 min, the association between IL-4Rα, Gab1, p-Gab1 and JAK1 was tested by co-immunoprecipitation assay (**H**). WT or Gab1 KO MEFs were treated with IL-4 (100 ng/mL) for applied time, tested by Western blotting of listed proteins (**J**). Quantification was performed from five replicate blot data (n=5, same for the blotting data in all Figures). Data were expressed as mean ± standard deviation (SD).*$P$< 0.05 vs. WT MEFs (**A**-**E**). [#] $P$< 0.05 vs. "Vector" (**E**). *$P$< 0.05 (**G**).

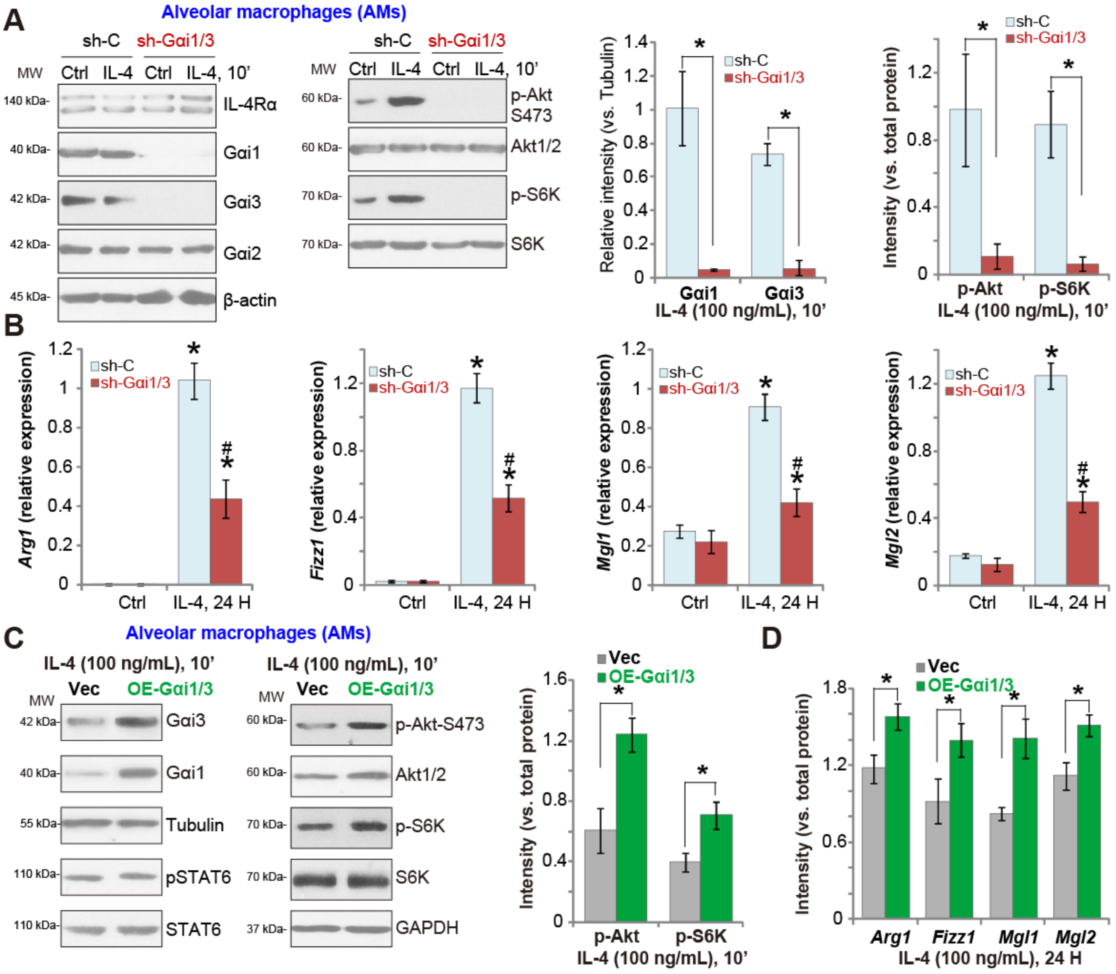


**Figure S2.** Alveolar macrophages (AMs), expressing the lentiviral scramble control shRNA ("sh-C") or the lentiviral Gαi1 shRNA plus Gαi3 shRNA ("sh-Gαi1/3") (**A** and **B**), the adenovirus Gαi1 construct plus the adenovirus Gαi3 construct ("OE-Gαi1/3") or empty vector ("Vec") (**C** and **D**), were treated with or without IL-4 (100 ng/mL) for 10 min, and tested by Western blotting of listed proteins (**A** and **C**). Twenty-four hours after IL-4 treatment, relative expression of listed genes was shown (**B** and **D**); In each experiment, n=5 (five replicated wells/dishes). Experiments were repeated five times (five mice per group), data of all repeated experiments were pulled together to calculate mean ±SD. ***$P$**< 0.05 (**A**, **C** and **D**). ***$P$**< 0.05 vs. "Ctrl" treatment in "sh-C"AMs (**B**). **#$P$**< 0.05 vs. IL-4 treatment in "sh-C"AMs (**B**).
